# The potential of glucagon-like peptide-1 receptor agonists in heart failure

**DOI:** 10.3389/fphys.2022.983961

**Published:** 2022-09-20

**Authors:** Frederik Flindt Kreiner, G. Kees Kornelis Hovingh, Bernt Johan von Scholten

**Affiliations:** ^1^ Global Chief Medical Office, Novo Nordisk A/S, Søborg, Denmark; ^2^ Department of Vascular Medicine, Amsterdam University Medical Center, Amsterdam, Netherlands

**Keywords:** heart failure, GLP-1, glucagon-like peptide-1, clinical trials, cardiovascular disease, cardiovascular outcome studies, diabetes, obesity

## Abstract

Heart failure (HF) remains one of the cardiovascular diseases (CVDs) associated with a high unmet medical need due to high morbidity and mortality rates and lack of efficacious interventions. HF is closely related to cardiometabolic diseases such as diabetes, obesity and chronic kidney disease, and strategies that address most or all these intertwined conditions are desirable. Glucagon-like peptide-1 receptor agonists (GLP-1 RAs) are approved for type 2 diabetes (T2D), and some are also indicated for reduction of the risk of atherosclerotic CVD in T2D and for weight management. As we summarise in this concise review, preliminary evidence suggests that the cardioprotective benefits of GLP-1 RAs may also extend to HF. The most robust clinical evidence arguably originates from the large cardiovascular outcomes trials (CVOTs) completed for most GLP-1 RAs, of which the latest showed a significant relative risk reduction (RRR) of 39% (HR) with once-weekly efpeglenatide on HF requiring hospitalisation, corroborating a meta-analysis which found a significant RRR across eight GLP-1 RA CVOTs of 11%. Further, although incompletely described, multiple studies are available to provide insights into the mechanistic underpinnings, which appear to be associated mostly with indirect cardioprotective benefits owing to the ability of GLP-1 RAs to address hyperglycaemia, and reduce body weight, and, amongst others, inflammation. In sum, current evidence positions GLP-1 RAs as a potential cardioprotective strategy in HF, with HF with preserved ejection fraction emerging as the clinically most relevant phenotype for the drug class, especially when occurring in people with obesity with and without diabetes.

## Introduction

Strategies to prevent or ameliorate heart failure (HF) remain urgently needed (Shah et al., 2020). Whereas pharmacotherapeutic and other interventions have steadily improved the prognosis of other types of cardiovascular disease (CVD) and associated sequelae, HF remains associated with more pronounced morbidity and mortality, constituting one of the most pronounced unmet needs in cardiovascular medicine (Shah et al., 2020). Furthermore, alongside the increasing prevalence of related cardiometabolic diseases such as diabetes, obesity and chronic kidney disease (CKD), the number of people with HF has increased to around 64 million globally, amongst whom most are also living with type 2 diabetes or obesity (Groenewegen et al., 2020). Considering their intertwined epidemiological and pathophysiological associations, interventions that simultaneously address many or all of these diseases are arguably desirable.

Within the past 2 decades, glucagon-like peptide-1 (GLP-1) receptor agonists (RAs) have emerged as effective agents to improve glycaemic control in type 2 diabetes and to reduce body weight in people with overweight or obesity (Müller et al., 2019; Nauck et al., 2020). In addition, three specific GLP-1 RAs are approved and recommended in several treatment guidelines to improve established CVD or CVD risk factors in people with type 2 diabetes (Arnett et al., 2019; Dunlay et al., 2019; Buse et al., 2020; Honigberg et al., 2020; Sattar et al., 2021; American Diabetes Association Professional Practice Committee, 2022). However, unlike for sodium-glucose co-transporter 2 (SGLT2) inhibitors (McDonagh et al., 2021; Heidenreich et al., 2022)–another novel drug class widely used in type 2 diabetes–a benefit for GLP-1 RAs in HF remains to be fully established in dedicated studies in people with type 2 diabetes as highlighted in some clinical guidelines (Dunlay et al., 2019). Nevertheless, reflecting theoretical and clinical evidence, other guidelines highlight the use of GLP-1 RA treatment for people with type 2 diabetes and HF (Buse et al., 2020; Cosentino et al., 2019), for example, the ADA/EASD consensus statement have hitherto recommended the use of GLP-1 RA treatment in situations where HF (and/or chronic kidney disease [CKD]) is predominant and SGLT2 inhibitors not tolerated (Buse et al., 2020).

In this article, we review current evidence suggesting a potential benefit of GLP-1 RAs in HF as well as the current understanding of the putative mechanistic underpinnings. Amongst the three predominant HF phenotypes (HF with preserved, mildly reduced or reduced ejection fraction; HFpEF, HFmrEF or HFrEF, respectively), we focus the review on HFpEF and HFrEF. Overall, results from the cardiovascular outcomes trials (CVOTs) completed for all marketed GLP-1 RAs in type 2 diabetes support a benefit in HF specifically in addition to the approved use in broad CVD risk reduction in people with type 2 diabetes (Sattar et al., 2021). However, additional trials are needed to increase the strength and granularity of the evidence in terms of, for example, safety considerations as well as benefits in people with comorbidities such as diabetes and obesity, and across HF phenotypes.

## The biology and pharmacology of GLP-1

**FIGURE 1:**
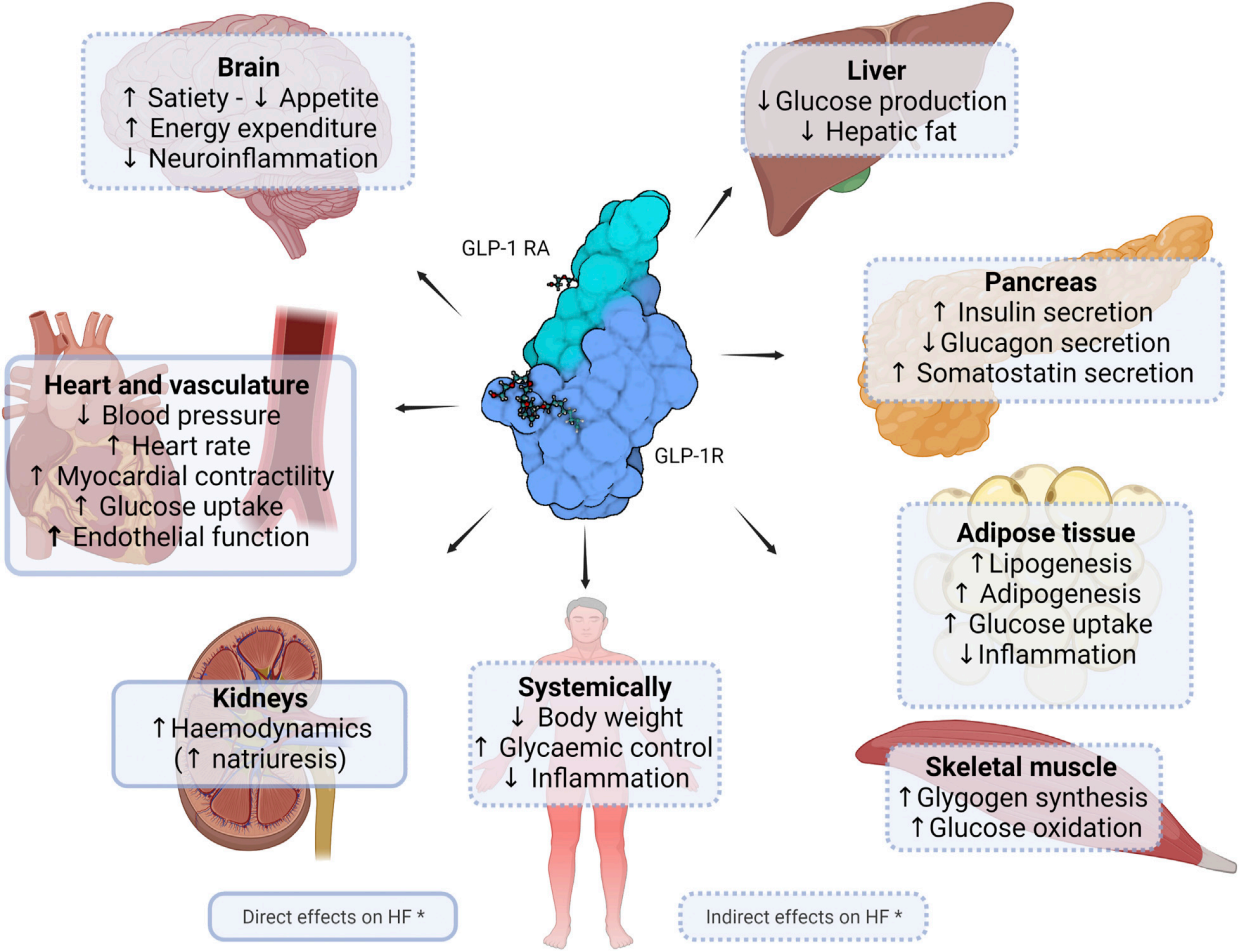
Potential direct and indirect effects of GLP-1 receptor agonists in heart failure. Figure shows currently known or suggested direct and indirect effects of glucagon-like peptide (GLP-1) receptor agonists of clinical relevance in heart failure. * Figure is not exhaustive and includes potential effects. Created with Biorender.com.

GLP-1 is a major peptide hormone of the incretin system. For an in-depth description of the biology of GLP-1, readers are referred to a recent review by Müller et al. (2019). Briefly, upon food intake, L cells in the intestine secrete GLP-1 into the circulation. The GLP-1 receptor (GLP-1R), which mediates the biological effects of the hormone with relevance for and beyond CVD, is present across many organs and tissues in humans (Figure 1). A major role of the hormone is to regulate the glucose homeostasis in a glucose-dependent manner as a result of its insulinotropic and glucagonostatic actions. GLP-1 effects in certain areas of the brain ultimately lead to weight loss, and in addition to the cardioprotective and renoprotective effects discussed in this article, evidence also points towards neuroprotective and other medically desirable properties (Müller et al., 2019; Nørgaard et al., 2022).

Currently, two main classes of GLP-1 RAs are available (Müller et al., 2019; Nauck et al., 2020): Compounds based on the lizard-derived exendin-4 peptide and compounds based on the human GLP-1 peptide. Both compound classes activate the human GLP-1R and have been engineered to have action profiles compatible with once-daily or once-weekly administration. Both classes are resistant to the enzymatic degradation seen for native GLP-1, which is cleaved by the DPP-IV enzyme to generate inactive metabolites and intact GLP-1 forms with low affinity for the GLP-1R. Most available GLP-1 RAs are given as subcutaneous injections. A second-generation GLP-1 RA (semaglutide) was introduced for oral administration as the world first’s large peptide in a tablet (Aroda et al., 2019). The safety and tolerability profiles of the GLP-1 RA drug class are consistent across indications and drug class members (Müller et al., 2019; Nauck et al., 2020). Side effects primarily include gastrointestinal events such as nausea, which can be mitigated using dose-escalation regimens that improve tolerance.

In accordance with their mechanism of action, all marketed GLP-1 RAs are indicated to improve glycaemic control in people with type 2 diabetes (Müller et al., 2019; Nauck et al., 2020). In addition, GLP-1 RAs liraglutide and semaglutide are indicated for weight management in people with obesity or overweight (Jensterle et al., 2022). Furthermore, CVOTs have documented the cardiovascular safety of the GLP-1 RA drug class in type 2 diabetes (Sattar et al., 2021); for some [dulaglutide (Gerstein et al., 2019), liraglutide (Marso et al., 2016) and semaglutide (Marso et al., 2017)], the CVOTs also confirmed a cardiovascular benefit and eventually led to the approved use of these GLP-1 RAs to reduce cardiovascular risk in people with type 2 diabetes and established CVD or at high cardiovascular risk (Honigberg et al., 2020). A recent meta-analysis by Sattar et al. (2021) of the CVOTs found that the GLP-1 RAs are associated with a significant 14% relative risk reduction in terms of major adverse cardiovascular events (MACE, a standard 3-component composite endpoint) (hazard ratio [HR] 0.86; 95%CI: 0.80–0.93) as well as in terms of the three individual components (cardiovascular death, myocardial infarction, and stroke). As discussed below, the CVOTs and the meta-analysis also indicated a benefit on HF.

## Epidemiology and pathophysiology of heart failure

As one of the most prevalent consequences of CVD and other cardiometabolic conditions such as diabetes and hypertension, HF remains associated with high mortality rates (Bragazzi et al., 2021). Estimates suggest that in 2017, approximately 64 million people world-wide suffered from varying degrees of HF (Groenewegen et al., 2020). In age-standardised numbers, the global prevalence was 831 individuals per 100.000 people (Bragazzi et al., 2021); despite a growing absolute number of cases, this represents a prevalence decrease of around 7% since 1990.

As outlined earlier, cardiometabolic diseases or risk factors such as type 2 diabetes (Nichols et al., 2004), obesity (Powell-Wiley et al., 2021) and hypertension (Oh and Cho, 2020) are major predictors for HF. Amongst people with HF, up to 50% are estimated to have type 2 diabetes and, vice versa, around 20% of the over 500 million people living with type 2 diabetes have been shown to have comorbid HF (Khan et al., 2020). Amongst people living with the HF with preserved ejection fraction phenotype (HFpEF; see below), 70% also live with obesity (Groenewegen et al., 2020) and 30% live with both type 2 diabetes and obesity (Lam et al., 2011) according to data from 2011. Accordingly, and in line with currently ongoing trials in HFpEF (ClinicalTrials.gov IDs NCT04916470 and NCT04788511), it may be relevant to consider obesity-related HFpEF as a specific (sub)phenotype, whether coexisting with type 2 diabetes or not.

Age is another major risk factor: HF has been reported to affect around 1%–2% of all adults (Groenewegen et al., 2020), rising to 10% in those over 75 years of age (Metra and Teerlink, 2017; Groenewegen et al., 2020; Weldy and Ashley, 2021). Further, in the Framingham Heart Study, almost all people with hypertension (91%) had or developed HF, the risk of which was 2-3-fold higher in hypertensive than in normotensive individuals (Vasan et al., 2001). Finally, CKD, including even mild kidney disease, is also associated with increased risk of developing CVD, including HF (Ataklte et al., 2021).

The pathophysiology of HF has been extensively reviewed by others (Metra and Teerlink, 2017; Weldy and Ashley, 2021). Briefly, HF refers to a malfunctioning heart that fails to pump blood at a sufficient rate relative to the demands of organs and tissues. Classification of HF is usually done by symptom severity or disease progression stage (McDonagh et al., 2021; Heidenreich et al., 2022). In the early stages of HF, compensatory mechanisms reduce the impact of the cardiac dysfunction; eventually, however, the progressive dysfunction results in clinical symptoms such as fatigue, reduced exercise capacity, dyspnoea, and congestion (“decompensation”). HF is directly related to the thickening and stiffening of the arteries as the results of, for example, atherosclerosis. Other common structural changes include aortic stenosis, myocardial fibrosis and myocyte stiffness. Eventually, these changes lead to increased peripheral resistance, left ventricular hypertrophy and atrial dilation. In addition, atrial fibrillation can occur, which, especially chronically, is associated with increased mortality.

As mentioned earlier, HF is traditionally considered as three main phenotypes: HFrEF, HFmrEF and HFpEF (Weldy and Ashley, 2021; Metra and Teerlink, 2017; Savarese et al., 2022). As implied by the terms, HFrEF represents HF where the left ventricular (LV) EF is markedly reduced (40% or lower), HFmrEF describes a mildly reduced LV EF of 40%–49%, whereas in HFpEF the LV EF is preserved at around 50% or more. HFpEF appears to be the most common type of HF, representing around 50% of the cases, with HFmrEF accounting for 10%–25% (Savarese et al., 2022). HFpEF is especially associated with age as well as female sex and conditions such as diabetes, obesity, hypertension, coronary heart disease and CKD.

Currently available pharmacotherapeutics options in HF mostly remain focused on HFrEF, where treatments strategies aim at relief of symptoms, improving quality of life, and prolonging the life of the affected individual (McDonagh et al., 2021; Heidenreich et al., 2022; Savarese et al., 2022). Mainstay treatments are renin-angiotensin-aldosterone system inhibitors, mineralocorticosteroid antagonists and β-blockers, and, as mentioned earlier, SGLT-2 inhibitors, especially in people with comorbid diabetes. Other options include vasodilators and diuretics. In HFpEF and HFmrEF, these drugs are also widely used, but their effect on clinical outcomes such as survival and hospitalisation is limited. In patients in whom the above-mentioned interventions are insufficient to control the manifestations of HF, additional mechanical circulatory support and/or cardiac transplantation may be indicated. A full overview of the indications of the specific treatments has been described in recent guidelines (McDonagh et al., 2021; Heidenreich et al., 2022).

## GLP-1 receptor agonism in heart failure

### Clinical evidence

Only a few clinical investigations studying the effects of GLP-1 RAs specifically in HF (all in HFrEF) have been reported as briefly discussed below. Accordingly, the bulk of the clinical evidence of the effects of the drug class on HF have been collected in the placebo-controlled CVOTs in people with type 2 diabetes and established CVD or CVD risk (Table 1) (Pfeffer et al., 2015; Marso et al., 2016; Holman et al., 2017; Marso et al., 2017; Hernandez et al., 2018; Gerstein et al., 2019; Husain et al., 2019; Gerstein et al., 2021). Integrating the result from the CVOTs in their meta-analysis, Sattar and colleagues found that the GLP-1 RA drug class may reduce the risk of hospital admission for HF by 11% (HR vs. placebo of 0.89, 95%CI 0.82–0.98) (Sattar et al., 2021). This reflects HRs <1 in all eight CVOTs except SUSTAIN 6 (HR 1.11, 95%CI 0.77–1.61), although statistical significance for this secondary outcome was shown for only efpeglenatide in AMPLITUDE-O (31) and albiglutide in HARMONY Outcomes, which also found the lowest HR (0.71, 95%CI 0.53–0.94) (Hernandez et al., 2018). This may suggest that the observation of a benefit of the GLP-1 RAs on heart failure hospitalisation as found in the meta-analysis by Sattar and colleagues is driven by data from AMPLITUDE-O and Harmony Outcomes. AMPLITUDE-O with efpeglenatide, the latest GLP-1 RA CVOT to report (Gerstein et al., 2021), indeed indicated a benefit on HF, with post hoc analyses showing a significant 39% relative risk reduction on HF requiring hospitalisation (HR 0.61; 95%CI 0.38–0.98) (Sattar et al., 2021). Further, whilst efpeglenatide was associated with a relative risk reduction of 30% in those who were not on SGLT2 inhibitors at trial entry (HR 0.70; 95%CI 0.42–1.17), for those who were treated with such agents at enrolment, the HR was as low as 0.23 and statistically significant in the post-hoc testing (95%CI 0.05–0.97) (Lam et al., 2021).

**TABLE 1:** Key cardiovascular outcomes results for selected GLP-1 receptor agonists.

GLP-1 RA (class, regimen)	CVOT (years of follow-up)	Population		Outcomes, hazard ratio (95%CI)		
		N	History of heart failure (established CVD^b^)	Hospitalisation for heart failure	3-point MACE^c^	Kidney outcomes (Sattar et al., 2021; von Scholten et al., 2022)
Efpeglenatide (exendin-4, s.c. OW^a^)	AMPLITUDE-O (31) (1.8 years)	4,076	18% (90%)	0.61 (0.38–0.98)	0.73 (0.58–0.92)	0.68 (0.57–0.79)
Lixisenatide (hGLP-1, s.c. OD^a^)	ELIXA (32) (2.1 years)	6,068	22% (100%)	0.96 (0.75–1.23)	1.02 (0.89–1.17)	0.84 (0.68–1.02)
Exenatide ER (exendin-4, s.c. OW)	EXSCEL (33) (3.2 years)	14,752	16% (73%)	0.94 (0.78–1.13)	0.91 (0.83–1.00)	0.88 (0.76–1.01)
Albiglutide (hGLP-1, s.c. OD^a^)	HARMONY(34) (1.5 years)	9,463	20% (100%)	0.71 (0.53–0.94)	0.78 (0.68–0.90)	N/A
Liraglutide (hGLP-1, s.c. OD)	LEADER (18) (3.8 years)	9,340	18% (81%)	0.87 (0.73–1.05)	0.87 (0.78–0.97)	0.78 (0.67–0.92)
Semaglutide, oral (hGLP-1, p.o. OD)	PIONEER 6 (35) (1.3 years)^d^	3,183	12% (85%)	0.86 (0.48–1.55)	0.79 (0.57–1.11)	N/A
Dulaglutide (hGLP-1, s.c. OW)	REWIND(17) (5.4 years)	9,901	9% (31%)	0.93 (0.77–1.12)	0.88 (0.79–0.99)	0.85 (0.77–0.93)
Semaglutide, s.c. (hGLP-1, s.c. OW)	SUSTAIN 6 (19) (2.1 years)	3,297	24% (83%)	1.11 (0.77–1.61)	0.74 (0.58–0.95)	0.64 (0.46–0.88)
Meta-analysis				**0.89 (0.82 to 0.98)** (Sattar et al., 2021)	**0.86 (0.80–0.93)** (Sattar et al., 2021)	**0.79 (0.73–0.87)** (Sattar et al., 2021)

CI, confidence interval; CVD; cardiovascular disease; CVOT, cardiovascular outcomes trial; GLP-1, glucagon-like peptide-1; hGLP-1, GLP-1, receptor agonist based on human GLP-1; MACE, major adverse cardiovascular event; OD, once-daily dosing; OW, once-weekly dosing; p.o., per oral administration in a tablet; RA, receptor agonist; s.c., subcutaneous injection.

a not marketed.

b trials enrolled people with established cardiovascular disease and/or elevated cardiovascular risk factors (see original publications).

c primary 3-point composite outcome (first occurrence of either cardiovascular death, myocardial infarction, or stroke; in ELIXA, also hospital admission for unstable angina).

d PIONEER, 6 was designed to document cardiovascular safety only.

It should be noted that evidence for HF specifically from the CVOTs are all associated with a number of limitations. First, none of the trials included HF as a component of the primary composite endpoint (usually 3-component MACE); many, however, stringently captured HF events as secondary endpoints. Nevertheless, for this reason alone, current evidence is not confirmatory. Second, although the CVOTs in general included a meaningful count of people with type 2 diabetes and HF at enrolment, the definition of HF was somewhat vague and varied across the trials; moreover, most were not analysed for outcomes based on presence of HF at enrolment. Third, none of the CVOTs discriminated between the HF phenotypes. Thus, whilst the CVOTs provide valuable and supportive evidence of a benefit of GLP-1 RAs on HF in people with type 2 diabetes, additional trials are required to fully understand the magnitude and nature of such a potential effect, including if it applies to both prevention and treatment of the condition.

As mentioned, trials focusing on GLP-1 RA treatment in HFrEF are few and of a smaller scale; further, those that have been reported in general showed a neutral or even seemingly detrimental effect of GLP-1 RA in this HF phenotype. Albiglutide was tested in an 82-person placebo-controlled trial but did not improve LVEF or other HF-related outcomes such as the 6-min walking distance test in people with type 2 diabetes and HF with LV EF <40% (Lepore et al., 2016). Liraglutide has been tested in two trials in people with HF and reduced LV EF. In the 24-week LIVE trial in people with stable chronic HF (with or without diabetes), liraglutide did not meaningfully improve LV EF or other variables including quality of life (Jorsal et al., 2017). Further, in the FIGHT trial, which was the only trial sufficiently comprehensive to allow for the evaluation of hard outcomes, even though the HR was above 1 for the composite endpoint of hospital admission due to HF, the treatment effect did not reach statistical significance (HR [liraglutide vs. placebo at 6 months] 1.3, 95%CI 0.89–1.88) in 300 people with HF, with and without type 2 diabetes and with recent decompensation (Margulies et al., 2016). The three trials in HFrEF are in themselves insufficient to conclude on the effects of GLP-1 RA treatment in this HF phenotype, and additional trials are therefore needed.

In HFrEF, an issue remains that because GLP-1 RAs have been shown to modestly increase heart rate (2-3 beats per minute) owing to their chronotropic effects (see “Mechanisms”) (Sun et al., 2015), GLP-1 RA treatment in HFrEF may be problematic considering that, in line with the use of beta-blockers to ameliorate HFrEF (11, 12), heart rate elevations may result in a worse outcome (DeVore et al., 2016; Ibrahim et al., 2019). In addition, although overweight and obesity is markedly more common, some individuals with advanced HF like HFrEF are also cachexic (Rossignol et al., 2015) and may therefore not benefit from the weight-reducing effect of GLP-1 RAs. These aspects likely need to be elucidated in future trials; however, to our knowledge, no larger clinical trials with GLP-1 RAs in HFrEF are ongoing.

### Mechanisms

The exact basis of the benefits on CVD risk of GLP-1 RA treatment (including the potential positive effects in HF) as discussed above remains incompletely elucidated.

Arguably, the most clinically relevant basis for the potential of GLP-1 RAs in HF could be their effect on multiple cardiometabolic parameters that play intertwined roles across the HF-related diseases such as diabetes and obesity, which often co-exist. Accordingly, GLP-1 RA treatment has been shown to directly or indirectly improve cardiometabolic risk factors that characterise diabetes and obesity, and which play central roles in the development or exacerbation of CVD, including HF. These factors include systemic inflammation (Bray et al., 2021; Zobel et al., 2021), hyperglycaemia (Mapanga and Essop, 2016), increased endothelial production of reactive oxygen species (ROS) (Ceriello et al., 2011; Li et al., 2017; Oh and Jun 2017; Lambadiari et al., 2018; Bray et al., 2021) and impaired vasodilation due to low nitrogen oxide bioavailability (Ceriello et al., 2011; Correale et al., 2021). As noted earlier, endothelial dysfunction from increased vessel thickness and stiffness due to for example atherosclerosis may induce or exacerbate cardiac dysfunction. GLP-1 RAs have been shown to improve endothelial function (Lambadiari et al., 2018), at least in part due to reduced atherosclerosis owing predominantly to the anti-inflammatory properties of the drug class (Rakipovski et al., 2018; Bray et al., 2021; Zobel et al., 2021) and perhaps to lowering of triglyceride levels (Hermansen et al., 2013; Song et al., 2015).

In concordance with the above, weight loss of a magnitude obtainable with bariatric surgery, or with newer agents such as semaglutide (Enebo et al., 2021; Wilding et al., 2021) or the GIP/GLP-1 dual receptor agonist tirzepatide (Rosenstock et al., 2021; Jastreboff et al., 2022), have been shown to be associated with lower risk of incident HF and to improve established severe HF (Sundström et al., 2017; Kindel and Strande, 2018; Aminian et al., 2020; Yang et al., 2020). Moreover, liraglutide 3.0 mg (i.e., the dose level approved for weight management for this GLP-1 RA) in combination with lifestyle intervention markedly reduced the presence of visceral fat over a period of 40 weeks (Neeland et al., 2021). It is especially this kind of adipose tissue that is believed to drive a large part of the pathological effects of excess body weight (Fox et al., 2007).

The reductions in ROS and systemic inflammation, in combination with improvements in the other cardiometabolic parameters, are also thought to mediate the substantiated but so far formally unconfirmed kidney-protective effects of GLP-1 RAs, which may also directly or indirectly carry a HF-related benefit, considering that CKD is a prominent risk factor for HF (von Scholten et al., 2022). Thus, in a recent meta-analysis (Table 1), the results of which resembles our and another similar recent analysis (von Scholten et al., 2022; Kristensen et al., 2019; Cha et al., 2021), Sattar et al. (2021) showed that the GLP-1 RA drug class may be associated with a relative risk reduction for clinical kidney outcomes of 21% (HR 0.79; 95%CI 0.73–0.87). The potential HF benefit associated with better kidney function may in part also be due to improved haemodynamic parameters that essentially relieve the failing heart of stress factors and workload. In this context, GLP-1 RA treatment has been shown to reduce diastolic filling pressures and other measures of diastolic performance (Saponaro et al., 2016; Bizino et al., 2019), although another study did not show such an effect, perhaps reflecting a shorter treatment period (Bojer et al., 2021).

Direct effects of GLP-1 receptor agonism on the heart is likely not a major contributor. This is in line with the fact that, although some studies have shown the presence of the GLP-1R in other parts of the heart (Wei and Mojsov, 1995; Wei and Mojsov, 1996; Ban et al., 2008), current evidence overall suggests that the receptor in humans is expressed predominantly in the sinoatrial node (Pyke et al., 2014; Richards et al., 2014). This understanding is in line with the chronotropic effects of GLP-1 RA treatment, which in a large meta-analysis was shown to be associated with heart rate increases of up to 3.35 beats/min (Sun et al., 2015).

Apart from the chronotropic effects, direct effects of GLP-1 specifically on the heart have not been comprehensively documented, although evidence exists to suggest that GLP-1 RAs may improve cardiac output as well as cardiomyocyte survival (Ravassa et al., 2012; DeNicola et al., 2014; Du et al., 2016; Zhang et al., 2016; Ma et al., 2020). GLP-1 has been shown to increase glucose uptake in the myocardium (Nikolaidis et al., 2005a; Zhao et al., 2006) and to improve LV function in dogs with dilated cardiomyopathy (Nikolaidis et al., 2005a), in which GLP-1 also attenuated reperfusion-related mechanical ventricular dysfunction not associated with irreversible myocardial damage (“myocardial stunning”) (Nikolaidis et al., 2005b). In line with the latter, GLP-1 have been shown to protect from ischaemia/reperfusion injuries in isolated perfused rat hearts (Bose et al., 2005). Taken together, additional studies are needed to fully clarify any cardioprotective effects of GLP-1 RA treatment directly on the heart.

It should be noted that one of the two major DPP-IV-generated truncated forms of GLP-1 (GLP-1 [9-36amide]) has been shown to have direct beneficial cardiac effects in mice, which is representative of the hypothesis that a GLP-1R-associated response can be elicited also by these metabolites and thus not only via the classic incretin pathway (Müller et al., 2019). However, in addition to a lack or ambiguity of similar findings in humans, GLP-1 RAs are resistant to enzymatic cleavage as noted earlier (Müller et al., 2019; Cherney et al., 2021). Thus, in the context of pharmacotherapeutic use of modern GLP-1 RAs and in terms of understanding the cardiovascular effects of such treatment, the biology of the truncated GLP-1 metabolites can be disregarded (Cherney et al., 2021).

## Future perspectives

In summary, the cardiovascular safety of all currently marketed GLP-1 RAs has been thoroughly established in large CVOTs (Sattar et al., 2021), of which some also suggested a substantial cardioprotective benefit. Accordingly, dulaglutide, liraglutide and semaglutide are indicated to reduce cardiovascular risk in people with type 2 diabetes. Further, the CVOTs in general also suggested a potential benefit of GLP-1 RAs on HF, positioning GLP-1 RAs as another advanced option alongside SGLT2 inhibitors in the otherwise sparse treatment armamentarium for especially HFpEF. Furthermore, smaller clinical trials as well as pre-clinical studies have provided some, albeit not conclusive, support for the clinical outcomes-based evidence of the benefit of GLP-1 RAs in HF. However, as discussed next, a bona fide indication for GLP-1 RAs to reduce the risk of developing HF or to treat chronic HF arguably requires the resolution of several unknowns.

As noted by others (Khan et al., 2020), it remains to be clarified if use of GLP-1 RAs is safe across the HF phenotypes. At present, the safety of the drug class in HFrEF needs to be further investigated (Khan et al., 2020). Thus, clinical trials are needed to establish the benefits/risk profile in HF, considering that no properly designed, HF-specific randomised clinical trials have been completed with GLP-1 RAs in any of the HF phenotypes. Whilst such trials are required to ensure medically appropriate adoption of the drug class in HF, they are also strongly warranted considering the current scarcity of pharmacotherapeutic options in HF (especially HFpEF), which remains one of the CVDs with markedly unaddressed medical needs. Further, whilst real barriers persist, available evidence has indeed corroborated the potential clinical utility of GLP-1 RAs in HF(5), with the most recent GLP-1 RA CVOT (Amplitude-O with once-weekly efpeglenatide) indicating a relative risk reduction for hospitalisation-requiring HF of at least 30% in people with type 2 diabetes (Gerstein et al., 2021).

Of note, the finding of a markedly greater benefit on HF of efpeglenatide amongst trial participants using SGLT2 inhibitors at enrolment in the AMPLITUDE-O CVOT highlights another important point (Lam et al., 2021). That is, investigations are warranted to better understand the potential benefit of combined use of GLP-1 RAs and SGLT2 inhibitors in HF with specific focus on the sequence of treatment initiation and the suitable population, including by HF phenotype and presence of diabetes. Combination with SGLT2 inhibitors to augment the benefits of this drug class in HFrEF with, for example, the weight-lowering effects of GLP-1 RA could offer a clinically valuable and feasible option addressing the spectrum of abnormalities associated with this HF phenotype.

Another drug class widely used to treat type 2 diabetes is inhibitors of the enzyme dipeptidyl peptidase-4 (DPP4), which mediates enzymatic breakdown of GLP-1. A study in rats (Ramírez et al., 2018) has showed that the DPP4 inhibitor sitagliptin induces a lowering of fatty acid utilization in cardiomyocytes in favour of glucose. This finding suggests that DPP4 inhibitors, owing to their GLP-1-enhancing actions, may have a direct beneficial effect on cardiomyocyte function. Conversely, data from clinical trials have showed an increased risk of incident heart failure with DPP4 inhibitors compared to placebo. This may be related to sympathetic activation by DPP4 inhibition (Packer, 2018). Overall, GLP-1 RAs appear to be more efficacious than DPP4 inhibitors in people with type 2 diabetes in terms of improving glycaemic control and reducing body weight (Gilbert and Pratley, 2020).

Yet another aspect to consider is the recurring theme of precision medicine. It is likely that more granular subphenotypes exist beyond the HFrEF/HFmrEF/HFpEF trichotomy. For example, an HFpEF phenotype characterised by pronounced expansion of epicardial adipose tissue (EAT) has been suggested (Elsanhoury et al., 2021). People with HFpEF with EAT might benefit specifically from EAT-directed intervention. To that end, GLP-1 RAs might be useful in light of studies demonstrating EAT reductions following treatment with dulaglutide, liraglutide or semaglutide (Yang et al., 2013; Beiroa et al., 2014; Iacobellis et al., 2017; Iacobellis and Villasante Fricke, 2020; Rasmussen et al., 2021). In addition, other patient-specific treatment strategies tailoring the use of GLP-1 RAs and other options to the HF phenotype and stage are likely also valuable to study and validate.

Finally, although most of the evidence of a potential benefit of GLP-1 RAs in HF originates from CVOTs in type 2 diabetes, it may likely be in the weight management setting that the drug class could be of the highest clinical value, particularly in people with HFpEF. In fact, as outlined above, HF is even more prevalent amongst people with obesity than amongst those with type 2 diabetes, and the consequences of excess body weight (i.e., adipose tissue) most likely play direct and indirect key roles in the development of HF. Accordingly, currently ongoing trials in HF (HFpEF) with the GLP-1 RA semaglutide (2.4 mg for once-weekly subcutaneous injection, i.e., the dose approved for weight management) enrols people with obesity with (STEP-HFpEF DM trial; Clinicaltrials.gov ID NCT04916470) or without (STEP-HFpEF trial; Clinicaltrials.gov ID NCT04788511) diabetes. In addition, the SUMMIT trial evaluates the effect of tirzepatide (the GLP-1/GIP dual receptor agonist) in people with obesity-related HFpEF (Clinicaltrials.gov ID NCT04847557).

## Conclusion

Taken together, it presently appears that amongst individuals with HF, those most likely to benefit from GLP-1 RA treatment are people living with or at risk of developing obesity-related (with or without diabetes) HFpEF. If in fact future studies robustly establish a beneficial effect of the drug class in HF, it may allow for broader adoption of GLP-1 RAs in the prevalent CVD, ultimately contributing to addressing the unmet medical need that continues to persist in the prevention or management of, in particular, HFpEF.
